# Novel heterozygous mutations of the SF1 gene and their functional characterization in patients with 46,XY disorders of sex development (DSD) without adrenal insufficiency

**DOI:** 10.1186/1687-9856-2015-S1-O53

**Published:** 2015-04-28

**Authors:** Jin-Ho Choi, Kyu Ha Woo, Ja Hye Kim, Ja Hyang Cho, Gu-Hwan Kim, Han-Wook Yoo

**Affiliations:** 1 Department of Pediatrics, Asan Medical Center Children’s Hospital, University of Ulsan College of Medicine, Seoul, Korea; 2 Medical Genetics Center, Asan Medical Center Children’s Hospital, University of Ulsan College of Medicine, Seoul, Korea

## Aims

Heterozygous mutations in SF1 have been identified in patients with 46,XY disorders of sex development (DSD) with normal adrenal function. This study was aimed to identify mutations in the SF1 gene in patients with 46,XY DSD and functional characteristics of their impact.

## Methods

This study included 48 patients with 46,XY DSD without adrenal insufficiency or dysmorphic features. Genomic DNA was extracted from peripheral blood leukocytes and direct sequencing of the 6 coding exons of SF1 was performed. To evaluate the effect of SF1 mutations on transcriptional activity, transient transfection studies were performed using dual luciferase reporter assay system with cotransfection of PGL4.75 Renilla luciferase as a marker of transfection efficiency. Wild-type, or mutant SF1 expression vectors with SF1 dependent StAR and MIS promoters linked to luciferase were assayed for luciferase activity.

## Results

Four of 48 patients (8.3%) harbored heterozygous novel sequence variants of the SF1 gene: p.G26A, p.C283R, p.L384RfsX7 and p.E445X. They presented female external genitalia with clitoromegaly in childhood or primary amenorrhea in adolescence. Endocrine evaluation at diagnosis showed low basal gonadotropin and testosterone levels. Functional studies of the p.G26A, p.C283R, and p.L384RfsX7 mutants demonstrated impaired transcriptional activation of SF1-responsive promoters. However, p.E445X mutant displayed no functional perturbation as wild-type.

## Conclusion

This study identified three novel loss-of-function mutations and their functional characteristics in patients with 46,XY DSD. Loss of function mutation in the SF-1gene is one of the relatively common causes of 46, XY DSD. Therefore, genetic defect of the SF-1 gene should be considered as an etiology in 46,XY individuals without adrenal insufficiency.

